# Aggressive Course of Myelin Oligodendrocyte Glycoprotein Antibody-Associated Disease (MOGAD): An Illustration of Two Cases and Review of Literature

**DOI:** 10.7759/cureus.68563

**Published:** 2024-09-03

**Authors:** Heitor C Frade, Awab Elnaeem, Pankhuri Banerjee, Tripti Sharma, Laura Wu, Alok Dabi

**Affiliations:** 1 Neurology, University of Texas Medical Branch, Galveston, USA

**Keywords:** myelin oligodendrocyte glycoprotein antibody-associated disease (mogad), cns inflammatory disorders, cns inflammatory demyelinating disease, acute disseminated encephalomyelitis (adem), myelin-oligodendrocyte glycoprotein antibody-associated disease, myelin-oligodendrocyte glycoprotein (mog), demyelinating autoimmune diseases

## Abstract

Myelin oligodendrocyte glycoprotein antibody-associated disease (MOGAD) is a central nervous system demyelinating disease that has become a major source of morbidity among children and adults. In the first case, we present an 18-year-old Hispanic female with a recently resolved upper respiratory infection who presented with fever, headache, progressive quadriparesis, urinary retention, and encephalopathy. The hospital course involved autonomic dysfunction and prolonged intubation requiring tracheostomy and gastrostomy. Cerebrospinal fluid (CSF) showed pleocytosis and a positive MOG titer (1:40). Magnetic resonance imaging (MRI) showed longitudinally extensive cervicothoracic T2 hyperintensity and brain multifocal T2 hyperintensities. After high-dose intravenous methylprednisolone (IVMP) and intravenous immunoglobulin (IVIG), she had full neurological recovery by the last follow-up. The second case is of a 22-year-old Hispanic male who presented with progressive lower extremity paresthesia and weakness over six weeks. CSF demonstrated pleocytosis, elevated protein, oligoclonal bands, and MOG antibody. MRI revealed multiple subcortical T2-hyperintense lesions and enhancing midcervical and lower thoracic lesions. Treatment with IVMP led to minor improvement with discharge on steroid taper and azathioprine. The patient’s disease progressed with a fluctuating course requiring two readmissions with upper extremity weakness, right optic neuritis, and urinary sphincteric dysfunction with neuroradiologic worsening. Treatment throughout multiple admissions included intravenous steroids, IVIG, plasmapheresis, mycophenolate mofetil, and rituximab with minimal improvement, symptom recurrence, and progression of multifocal lesions. The patient died four months after the symptom onset.

These cases had markedly different treatment responses despite similar baseline characteristics. The difference in morbidity and disability burden highlights the importance of further investigation of this condition through clinical trials.

## Introduction

Myelin oligodendrocyte antibody-associated disease (MOGAD) is an important differential diagnosis to consider when evaluating central nervous system demyelinating conditions. There are limited epidemiological data on the US population, with an estimated prevalence of 0.16-0.48 per 100,000 patient-years in European children and adult populations, with similar gender distribution in both groups, but is more prevalent in children [1-3].

MOGAD was initially considered a subtype of neuromyelitis optica spectrum disorders (NMOSD) due to overlap in its clinical presentation; however, differences in immunopathogenesis and clinical characteristics eventually led to the dissociation between the two entities.

The first suggestion for antibodies targeting myelin sheath from oligodendrocytes emerged in 1976 when Lebar et al. found a complement-fixing autoantibody causing demyelinating activity in experimental allergic encephalomyelitis (EAE) [4]. Subsequent EAE models identified immunogenic particles specific to central nervous system (CNS) myelin were found to be associated with demyelinating lesions, such as 8-18C5 antibodies by Linnington et al. [5], and M2 antigen identified in later work of Lebar et al. [6]. It was not until 1987 that myelin oligodendrocyte glycoprotein (MOG) antibodies were described and found to be correlated as serum demyelinating markers [7].

MOG antibodies detected through enzyme-linked immunosorbent assay or western blot were soon found to be associated with some forms of multiple sclerosis (MS), though with lower sensitivity and specificity [8,9]. MOG antibodies (MOG-Ab) were also associated with NMOSD, particularly in cases of bilateral optic neuritis [10]. Cell-based assays for MOG-Ab improved sensitivity and specificity for demyelinating conditions, and a large subset of patients of NMOSD was found to be associated with MOG antibodies, exclusively when aquaporin (AQP)-4 antibodies were negative [11,12].

Patients with AQP-4 negative NMOSD and MOG-Ab positive were still considered a subtype of AQP-NMOSD due to considerable clinical overlap. As opposed to multiple sclerosis (MS), both entities commonly present with bilateral optic neuritis and longitudinally extensive transverse myelitis (LETM), including gray and white matter involvement. In a nationwide French cohort of 197 patients with an incident or retrospective diagnosis of MOG-Ab-positive acquired demyelinating syndromes, only 19.3% met diagnostic criteria for NMOSD and 1.5% for MS at the last follow-up, which precipitated the separation of MOGAD as an entity of its own [13,14]. With further understanding of the clinical, laboratory, and radiologic features of MOGAD, an international MOGAD panel has proposed new diagnostic criteria to differentiate it from NMOSD and multiple sclerosis [15].

The clinical presentation can include optic neuritis, longitudinally extensive transverse myelitis (LETM), acute brainstem syndrome, acute disseminated encephalomyelitis (ADEM), and multiphasic disseminated encephalomyelitis (MDEM). The clinical spectrum of MOGAD may vary from an ADEM-like presentation (including ADEM, ADEM-optic neuritis, and MDEM), more common in children below nine years of age, to an optic-spinal syndrome (optic neuritis, myelitis, and brainstem encephalitis), more prevalent in adolescents and young adults.

Short-term treatment for different clinical MOGAD phenotypes is based on the initial clinical presentation, as anti-MOG antibody (Ab) testing results may take days. Like other demyelinating diseases, high-dose intravenous methylprednisolone (IVMP) is the mainstay of acute treatment for MOGAD. More severe acute presentations may also benefit from intravenous immunoglobulin (IVIG) and plasma exchange (PLEX). Maintenance therapy is typically indicated in individuals who have two or more attacks but can be selectively used in patients with residual severe symptoms from a single attack. The most common medications used for maintenance are monthly IV immunoglobulin (IVIG) infusion, rituximab, azathioprine, mycophenolate, and IL-6 inhibitors.

There is no consensus regarding the duration of long-term treatment of ADEM-like MOGAD, given the monophasic pattern of ADEM. Since this MOGAD variant has a higher relapse rate than classical ADEM, some experts use rituximab or other immunosuppressants based on the treatment of other MOGAD phenotypes. MOG-Ab seroconversion guides this decision for some clinicians, but patients may revert to seropositivity after negative antibody testing following MOGAD treatment. Outcomes in MOGAD are usually better than in NMOSD, but 45% of treated patients have residual severe disability, and up to 70% of patients have one or more relapses, especially during steroid weaning or within two months of steroid discontinuation [16].

In this study, we discuss two patients with MOGAD, one with ADEM-phenotype and another with NMOSD-like phenotype, who were admitted to our service and had a markedly different clinical course, response to treatment, and outcome.

## Case presentation

Case 1

We present an 18-year-old Hispanic female who had a prolonged ICU admission for severe encephalomyelitis (ADEM-phenotype MOGAD) that resolved within three months. She was previously healthy and had recently received routine college admission vaccination one month prior to the presentation. She initially developed fever, headache, neck pain, and bilateral lower extremity numbness and weakness that progressed rapidly over two days, leading to her inability to walk.

Shortly after the presentation, she developed reduced consciousness, hypotension, and oxygen desaturation, requiring intubation. While intubated, examination revealed intact cranial nerve function, including blink to threat. Her strength was 2/5 in the upper extremities and 0/5 in the lower extremities, with urinary retention.

**Figure 1:**
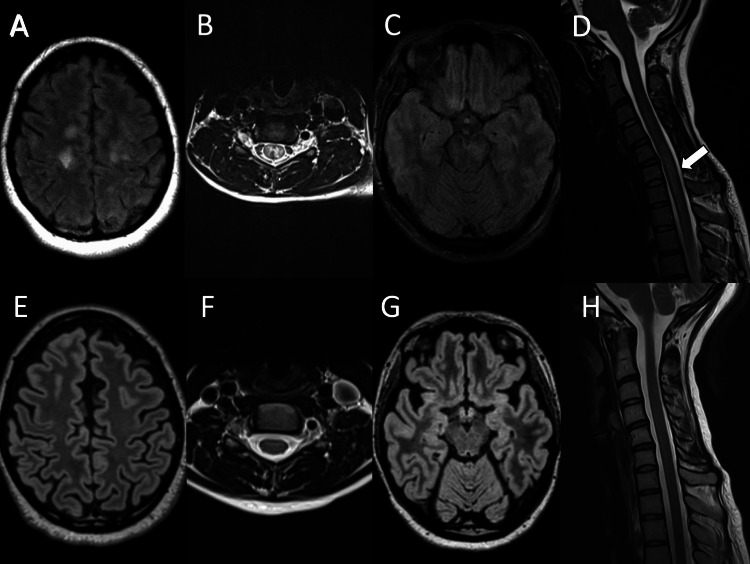
MRI brain and cervical spine from Case 1 at initial presentation. FLAIR MRI, axial view, showing subcortical frontal hyperintense foci (A); T2 MRI, axial view, of cervical spine showing hyperintense grey matter, H-sign (B); FLAIR MRI, axial view, showing left midbrain involvement (C); and T2 MRI, sagittal view, of cervical spine, arrow showing hyperintense cord signal (D). Interval resolution of T2-hyperintense lesions on subcortical frontal lobes (E), cervical spine (F), midbrain (G), and cervical spine on follow-up imaging three months later (H). MRI: magnetic resonance imaging; FLAIR: fluid-attenuated inversion recovery image

Testing on day one showed normal hemogram, routine blood chemistry, negative tests for fecal pathogens, HIV, COVID-19, and serum acetyl-choline esterase and antinuclear antibody. Spinal tap analysis revealed a protein level of 45 mg/dL, glucose level of 45 mg/dL, and a white blood cell count of 128 cells/µL (74% lymphocytes), with a negative meningitis-encephalitis panel and no detectable anti-NMDA antibodies. MRI of the brain and spine (day one) showed diffuse T2-hyperintense lesions involving cortical, subcortical, deep white matter areas, the brainstem, and the spinal cord, with no contrast enhancement (Figures 1A-1H).

She was started on IV methylprednisolone (IVMP) 1 g daily for five days, followed by rapid taper with oral prednisone to maintain a dose of 20 mg daily. Given the lack of initial improvement with IVMP, she additionally received a course of intravenous immunoglobulin (IVIG) between post-admission days two and six.

Test results were positive for elevated myelin basic protein (day two, >106; normal range (NR): 0-5.5), IgG index (day three: 0.75; NR: 0.28-0.66), MOG antibody (Ab) (day 12: 1:40; NR: <1:10), and normal oligoclonal bands (day one: 0 bands) and AQP-4 Ab (day five: <1.5; NR: <2.9) (Table 1).

**Table 1:** Serum and cerebrospinal fluid laboratory findings. CSF: cerebrospinal fluid; WBC: white blood cell; RBC: red blood cell; EBV: Epstein-Barr virus; AFB: acid-fast bacillus; ACE: angiotensin-I-converting enzyme; NMDAR: N-methyl-D-aspartate receptor; MBP: myelin basic protein; OCB: oligoclonal bands; IgG: immunoglobulin G; MOG: myelin oligodendrocyte glycoprotein; CBA-IFA: cell binding assay-immunofluorescence assay; HIV: human immunodeficiency virus; HTLV: human T-lymphotropic virus; RPR: rapid plasma reagin; QTB: QuantiFERON-TB Gold; ANA: antinuclear antibody; CRP: C-reactive protein; ESR: erythrocyte sedimentation rate; AQP4: aquaporin-4; ELISA: enzyme-linked immunoassay; PCR: polymerase chain reaction

Variables	Case 1	Case 2 (first admission)	Case 2 (day 63)	Reference values
CSF				
Protein (mg/dL)	77	62	-	15-45
Glucose (mg/dL)	53	102	-	40-70
WBC (1,000 cells/μL)	144	32	-	0-4
Lymphocytes	67%	91%	-	30-90%
Neutrophils	26%	-	-	0-6%
RBC (1,000 cells/μL)	29	6	-	0-1
EBV quantitative PCR	Not detected	Not detected	-	Not detected
AFB culture	Negative	-	-	Negative
West Nile IgG by ELISA (IU)	0.1	0.35	-	<1.29
Coxsackie A antibodies	<1:1	-	-	<1:1
Coxsackie B (1-6) antibodies	<1:1	-	-	<1:1
Echovirus antibodies	<1:1	-	-	<1:1
ACE (U/L)	0.9	1.1	-	0.0-2.5
Anti-NMDAR IgG antibody	<1:1	-	-	<1:1
MBP (ng/mL)	>106	>106	-	0.0-5.5
OCB	0	6	-	0-2
IgG index	0.75	0.66	-	0.28-0.66
MOG Ab by CBA-IFA		1:8	-	<1:2
Serum				
WBC (1,000 cells/μL)	12.5	6.7	-	4.5-12.5
Lymphocytes	8.4%	27.6%	-	24-44%
Neutrophils	85.4%	65.3%	-	36-66%
RBC (1,000 cells/μL)	13.7	5.64	-	4.04-5.86
Platelets (1,000 cells/μL)	327	295	-	150-400
Vitamin B12 (pg/mL)	-	308	-	240-930
Vitamin D, 25-OH (ng/mL)	<13	-	-	25-80
HIV 1/2 Ag-Ab	Negative	Negative	-	Negative
HTLV I/II antibodies by ELISA		Negative	-	Negative
RPR	-	Nonreactive	-	Nonreactive
West Nile IgM by ELISA (IU)	0.04	-	-	<0.89
Mycoplasma IgM by ELISA (U/L)	0.4	-	-	≤0.76
QTB assay	-	-	Negative	Negative
Lyme antibodies by ELISA (LIV)		-	0.12	0.0-1.2
ACE (U/L)	22	-	-	16-85
ANA	Negative	-	-	
CRP (mg/dL)	-	0.3	-	<0.8
ESR (mm/h)	-	2	36	0-10
AQP4-Ab (U/mL)	<1.5	<1.5	-	≤2.9
MOG IgG by CBA-IFA	1:40	-	<1:10	<1:10

Clinically, she continued to deteriorate for the first week with only intact visual tracking and blink to command and 0/5 strength in all extremities on day six. She improved slowly and was able to flex and extend her neck by day eight, bend her elbows by day 12, move her legs against gravity on day 17, regain 4/5 strength of all extremities by day 28, and was discharged on day 37 with 5/5 limb strength. Maintenance azathioprine 100 mg daily was added to prednisone on discharge.

She was seen in the clinic on day 69 where she ambulated independently with residual mild paresthesia. At a three-month clinic follow-up, she was normal on neurologic examination, and the MRI brain and spine were normal without evidence of lesions (Figures 1A-1H). MOG Ab titer was negative (<1:10, NR: <1:10) (Table 1).

Case 2

A 22-year-old Hispanic male presented with rapidly progressive lower extremity weakness (NMOSD-phenotype), followed by frequent relapses of multifocal neurological dysfunction. Despite aggressive treatment, he died within four months of initial presentation. He was previously healthy and had no history of prior trauma, infectious illness, or immunizations. There was no family history of immunologic or neurologic disease. He lived with his mother and stepfather.

He initially presented with three weeks of progressive bilateral lower extremity tingling and numbness, and three days of bilateral lower extremity weakness, with difficulty in standing for an extended time. Examination showed normal cranial nerve function, including visual acuity, 5/5 upper extremity strength, 4/5 lower extremity strength, 3+ deep tendon reflexes in all extremities, positive Hoffman’s sign bilaterally, and mute plantar responses. Sensory examination revealed hyperesthesia of the dorsal midthoracic area and reduced pinprick and vibration of the lower extremities.

**Figure 2:**
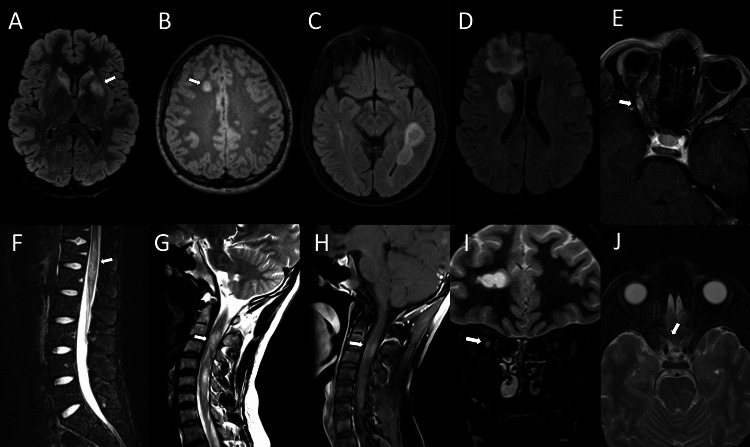
MRI brain and spine from Case 2 at initial presentation to our service. FLAIR MRI, axial view, showing T2-hyperintense lesions involving left caudate (A), right frontal corona radiata (B), left temporal lobe (C), right frontal lobe (D), and right optic nerve sheath (E). T2/short-tau inversion recovery MRI, sagittal view, showing T2-hyperintense conus medullaris (F). Sagittal cervical spinal cord with T2-hyperintense signal (G), associated with enhancing signal on sagittal T1 post-contrast (H). T2 MRI, coronal view, showing right optic nerve hyperintense rim (I). T2 MRI, axial view, showing T2 hyperintensity on right optic nerve sheath (J). MRI: magnetic resonance imaging; FLAIR: fluid-attenuated inversion recovery image

Laboratory testing (day one) showed a normal hemogram, routine blood chemistry, B12 level, folate level, thyroid panel, and glycated hemoglobin. He tested negative for HIV and COVID-19. Magnetic resonance imaging (MRI) of the spine (day three) showed fluid-attenuated inversion recovery (FLAIR) hyperintensities associated with contrast enhancement in C3-6, T12, and the conus medullaris. MRI brain (day three) showed noncontrast-enhancing FLAIR hyperintensities involving bilateral basal ganglia and periventricular areas (Figures 2A-2J). He was treated with intravenous methylprednisolone (IVMP) at 1 g/day for five days (days three to seven). Cerebrospinal fluid (CSF) studies (day four) showed elevated protein, normal glucose, and lymphocytic pleocytosis with 91% lymphocytes.

On day eight, a repeat CSF test showed a normal IgG index of 0.66 (NR: 0.28-0.66). The patient was started on azathioprine 50 mg daily and a six-day oral methylprednisolone taper dose with a diagnosis of atypical demyelinating disease. After discharge, he still demonstrated elevated myelin basic protein (MBP) and normal AQP-4 Ab <1.5 U/mL (NR: <2.9 U/mL) on day 10, and elevated MOG-Ab titer at 1:8 (NR: <1:2) on day 18 (Table 1).

He was readmitted on day 16 with a new right eye blurry vision while reporting improved leg weakness. The visual acuity examination showed significant impairment in the right eye (<20/200) with an intact ability to count fingers, with normal acuity in the left eye. MRI brain showed new contrast-enhancing lesions associated with FLAIR hyperintensities in the right frontal lobe and optic nerve. MRI spine showed no new lesions.

He received intravenous methylprednisolone at 1 g/day between days 18 and 22, followed by an extended taper over 18 days, then a maintenance dose of oral prednisone 10 mg daily. Additionally, he received intravenous immunoglobulin (IVIG) of 0.5 g/kg over four days due to lack of improvement after the steroid therapy. By discharge (day 28), his visual acuity subjectively improved but remained at 20/200. His upper and lower extremity strength improved by two points each on the motor scale.

Five weeks later, he was readmitted with recurrent right eye blurred vision and motor weakness of limbs. Examination showed visual acuity of the right eye at 20/100 and the left eye at 20/30. Strength in the right upper extremity was 3/5 and in the rest of the limbs 4/5. MRI brain showed new left frontotemporal lesions, bilateral brainstem, and basal ganglia lesions. MRI spine showed disease progression involving segments C2-7 and T5-10. He was treated with plasma exchange, a total of five sessions (days 68-72). His examination was unchanged by discharge (day 73). Further treatment with rituximab was planned, but the patient preferred to return home while waiting for insurance approval.

He was readmitted on day 76 for urinary retention with urinary tract infection (UTI) and was treated with antibiotics. Episodic tachycardia of unclear etiology was observed during that admission. The patient was discharged with an indwelling bladder catheter. He visited the emergency department a month later for recurrent UTI and was treated with outpatient antibiotics.

The patient started receiving rituximab on day 109. Two weeks later, when he was supposed to receive a second rituximab infusion, his mother called the hospital to report he passed away during his sleep on day 123.

## Discussion

MOGAD is a rare, recently recognized, unique neurologic demyelinating disorder with a wide range of clinical phenotypes and treatment responses. Although there have been unifying efforts in diagnosis and management, there are heterogeneous presentations and responses to treatment, even in individuals with similar baseline characteristics. 

The two patients reported here were both Hispanic young adults born and raised in the Southern United States with no past medical or family history of autoimmune disorders. One had been recently exposed to college preadmission immunizations, while the other had no apparent triggers leading to disease onset. The 1:1 gender ratio described here is representative of what has been previously reported for MOGAD, in contrast to approximately 3:1 in MS and 9:1 in NMOSD. Ethnicity has not been found to be associated with the prevalence or severity of MOGAD [17].

While the first patient had a more aggressive presentation with encephalopathy and quadriparesis (ADEM-phenotype) and required prolonged intubation and tracheostomy, she went on to have near-total recovery over the subsequent four months, with full resolution of motor and sensory symptoms but residual neurogenic bladder. The second patient had several relapsing presentations without any encephalopathic symptoms, affecting optic nerves and limb motor function (NMOSD-phenotype), with incomplete recovery after each episode, and the patient ultimately died after four months.

During the first hospitalization of Case 2 at an outside facility, when MOG antibodies were first detected, he received a five-day course of pulse-dose steroids with rapid taper and was discharged on azathioprine. The rapid taper of steroids could have contributed to an earlier relapse, 16 days post-discharge with progressive symptoms. Steroid taper has been associated with a 59% relapse rate when tapered over less than one month and a 47% relapse rate in less than three months [18,19].

While young patients tend to have a monophasic clinical syndrome with ADEM, more recent case reports indicate a recurrence within five years with other clinical phenotypes of MOGAD [20]. There is still a paucity of evidence to predict relapses in MOGAD. In a single-center cohort of 48 patients who had relapsing course, Hispanic or non-Hispanic nonwhite was the only baseline characteristic associated with a significantly higher risk of relapse when adjusted for age at index event, sex, and MOGAD clinical phenotype [21].

Relapses have been more common in patients who received treatment for less than three months, as well as in patients with persistently elevated MOG antibody titers. Case 2 was found to have undetectable MOG antibodies at his second relapse. Although uncommon, MOGAD relapses have been reported in 14.3% of prospectively followed cases in a Spanish center [11]. Relapse may also be more frequent in individuals with co-occurrence of CSF oligoclonal bands and MOG-IgG antibodies, which was the serology profile of our second patient [22].

Given the quickly progressive course of Case 2, following his second relapse, he was treated with plasma exchange (PLEX), with subsequent stabilization of symptoms. He was started on mycophenolate mofetil and discharged. Five days later, the patient was readmitted after a relapse of optic neuritis. Given the poor response to multiple acute immunomodulating treatments, rituximab outpatient infusion was requested. Four weeks after that third relapse, he received the first of two doses of rituximab 20 mg/kg before his death on the day that the second rituximab dose was scheduled, four months after symptom onset.

A 1999 randomized double-blinded trial that used plasma exchange (PLEX) for acute inflammatory demyelinating conditions, including MS and non-MS demyelinating disorders, was found to have a significant symptomatic improvement in patients with optic neuritis, transverse myelitis, and ADEM. Most of the demyelinating illness patients present acutely and can end with high disease burden and morbidity if not treated early [23].

As with many other immunomodulating therapies, the rationale for rituximab use in MOGAD has been derived from its efficacy in NMOSD. Although more modest than in NMOSD, decreases in relapse rates ranging from 30% to 42% have been reported [24,25]. The limitation in rituximab efficacy may be related to the absence of MOG-specific B cell frequency in 40% of MOGAD patients [26].

In a multicenter cohort, rituximab has fared better than mycophenolate mofetil and is like azathioprine in preventing MOGAD relapse. IVIG had the best relapse rate on follow-up, with no relapse among patients who received it as the first maintenance therapy, while relapse occurred in 1/3 of the remaining 60% of patients who received IVIG as second or later maintenance therapy [27]. Our second patient never received maintenance IVIG although a partial response was reported during the acute treatment of his first relapse.

In a short case series, patients with a severe MOGAD presentation that was incompletely responsive to standard IV steroids followed by IVIG or PLEX had marked improvement after tocilizumab treatment [28]. Tocilizumab may also be effective in cases that had a refractory course despite the use of rituximab as maintenance therapy, as several groups have now reported [29].

The patient died during his sleep on day 123 of symptoms. The circumstances preceding this patient's death were not entirely clear. On his last hospital admission, he was found to have urinary retention and symptomatic tachyarrhythmia both suggesting dysautonomia. The cause of death remains unclear, but these findings suggest that the patient was vulnerable to tachyarrhythmias, a potentially fatal complication.

There are limitations to our case reports that may impact generalizability. First, although the clinical complexity and refractory course of Case 2 may provide insight into treatment-resistant cases, he did not receive an extended steroid taper lasting over a month, as currently supported by the literature. Second, we do not have a clear understanding of the preceding events that led to his death, and autopsy was not permitted by the family.

The cases illustrated further highlight that, even in similar sociodemographic strata, the severity of presentation, MOG antibody titers, type of clinical phenotype, and use of rapid immunomodulating strategies may still lead to different clinical courses and long-term outcomes.

## Conclusions

Although MOGAD presentations have been recently established, management and outcomes are still heterogeneous. The distinct responses to the treatment described here and the limited applicability of many treatments targeted to MS and NMOSD highlight the importance of prospective studies, including randomized clinical trials on this condition.
